# Phytoplankton community responses to sub-inhibitory levels of four contaminants of emerging concern

**DOI:** 10.1093/plankt/fbag072

**Published:** 2026-08-26

**Authors:** Catherine Schlenker, Gavin Madgett, Henry Guy, Tammi L Richardson, Eilea Knotts, James L Pinckney

**Affiliations:** Department of Biological Sciences, University of South Carolina, 715 Sumter St, Columbia, SC 29208, USA; School of the Earth, Ocean, and Environment Marine Science Program, University of South Carolina, 701 Sumter St, Columbia, SC 29208, USA; School of the Earth, Ocean, and Environment Marine Science Program, University of South Carolina, 701 Sumter St, Columbia, SC 29208, USA; Department of Biological Sciences, University of South Carolina, 715 Sumter St, Columbia, SC 29208, USA; School of the Earth, Ocean, and Environment Marine Science Program, University of South Carolina, 701 Sumter St, Columbia, SC 29208, USA; Institute for Clean Water and Healthy Ecosystems, University of South Carolina, Columbia, SC 29208, USA; Department of Biological Sciences, University of South Carolina, 715 Sumter St, Columbia, SC 29208, USA; Department of Biological Sciences, University of South Carolina, 715 Sumter St, Columbia, SC 29208, USA; School of the Earth, Ocean, and Environment Marine Science Program, University of South Carolina, 701 Sumter St, Columbia, SC 29208, USA; Institute for Clean Water and Healthy Ecosystems, University of South Carolina, Columbia, SC 29208, USA

**Keywords:** Chemtax, Bayesian, breakpoint, aquatic, phytoplankton

## Abstract

Contaminants of emerging concern (CECs) are increasingly prevalent in freshwater systems, yet their effects on phytoplankton communities remain poorly understood. We examine how a representative freshwater assemblage responds to four ubiquitous CECs [carbamazepine, diclofenac, 1H,1H,2H,2H-perfluorooctanesulfonic acid (6:2 FTS) and 6ppd-quinone (6PPD-Q)] at environmentally relevant concentrations, monitoring changes in chlorophyll *a* (chl *a*), taxonomic composition and photosynthetic efficiency. Bayesian segmented regression (BSR) identified pollutant-specific ecological thresholds (breakpoints). Phytoplankton responses varied markedly among contaminants. 6:2 FTS showed the most consistent above- and below-breakpoint directional slope patterns; most algal groups were modestly stimulated at low concentrations, declining above the threshold. Diclofenac showed tight clustering of algal group breakpoints. Carbamazepine showed dispersed breakpoints, with several sensitive taxa responding above the community-level threshold. 6PPD-Q produced the highest and most variable breakpoints, with significant early declines in chl *a*. These results suggest that phytoplankton sensitivity to CECs is strongly influenced by contaminant-specific mechanisms and taxa-specific physiology. Chemicals with consistent effects (e.g. 6:2 FTS) can be monitored using community-level metrics, whereas compounds with heterogeneous thresholds (e.g. carbamazepine and 6PPD-Q) require multi-taxa monitoring to detect early ecological changes. Applying BSR to natural communities provides a robust framework for assessing sub-inhibitory impacts of contaminants, thereby enhancing ecological risk assessments.

## INTRODUCTION

Contaminants of emerging concern (CECs) have become an increasingly important research focus, examining how biologically and chemically derived pollutants from human activity affect aquatic ecosystems. CECs include pharmaceuticals and personal care products, tire wear particles and persistent organic pollutants, among others, that may enter surface water via stormwater runoff, sewage, wastewater and industrial sources. Their persistence, ubiquity and documented physiological impacts on some aquatic organisms have raised global concern (Boxall *et al*., 2012). Although there have been extensive toxicological studies on monocultures of algal species, the responses of natural phytoplankton communities remain insufficiently described. Characterizing CEC exposure responses in natural phytoplankton communities at closer to environmentally relevant concentrations is necessary to assess realistic species interactions and understand how environmental histories influence community responses. This study investigated the impact of four contaminants, carbamazepine, diclofenac, 1H,1H,2H,2H-perfluorooctanesulfonic acid (6:2 FTS) and 6ppd-quinone (6PPD-Q), which vary in classification and use, on a natural freshwater phytoplankton community.

The pharmaceutical carbamazepine is an antiseizure drug used to treat epilepsy and other seizure disorders, as well as some mental health conditions (Alrashood, 2016). This CEC is among the most ubiquitous pharmaceuticals in global surface waters because carbamazepine is highly resistant to degradation and typical water treatment processes (Radjenović *et al*., 2009; Chia *et al*., 2021). Carbamazepine may inhibit the growth of phytoplankton species, including *Scenedesmus obliquus* and *Chlorella pyrenoidosa*, at higher concentrations. However, it is generally regarded as having low phytotoxicity and high EC_50_ values, indicating relatively little impact on phytoplankton community biomass (DeLorenzo and Fleming, 2008;Zhang *et al*., 2012; Jarvis *et al*., 2014).

Another CEC frequently measured in the environment is diclofenac, a non-steroidal anti-inflammatory drug used to treat arthritis and other soft-tissue injuries (Banning, 2008). Like carbamazepine, diclofenac is resistant to degradation and water treatment processes, making it among the most frequently detected pharmaceuticals in surface water (Radjenović *et al*., 2009; Chia *et al*., 2021). Sha’aba *et al.* (2025) suggest that exposure to diclofenac may inhibit phytoplankton growth and reduce diversity, and there is evidence that it may inhibit the growth of monocultures of the chlorophyte *Pseudokirchneriella subcapitata* (Hernández-Zamora *et al*., 2025).

The “forever chemical” 6:2 FTS is part of the per- and polyfluoroalkyl substances (PFAS) group, which are widely known and used for their hydrophobicity, oleophobicity and low reactivity, making them well-suited for many industrial applications (Glüge *et al*., 2020). PFAS are typically found at higher concentrations near industrial sources and in urbanized areas, but the fluorinated chains characteristic of PFAS, including 6:2 FTS, confer stability in water and thus widespread distribution in aquatic ecosystems (Jarvis *et al*., 2021; Tang *et al*., 2023). 6:2 FTS is being used more frequently as a substitute for longer-chain PFAS that have been phased out of production due to their adverse environmental and public health impacts (Blake *et al*., 2024). There is evidence that it is less toxic than perfluorooctane sulfonate (PFOS) to the green algae *Chlorella vulgaris* and *P. subcapitata* and the cyanobacteria *Microcystis aeruginosa*; however, it remains understudied relative to the longer-chain PFAS, such as PFOS (Zhang *et al*., 2023).

The contaminant 6PPD-Q is a degradation product of 6ppd, an antiozonant used to enhance tire longevity (Krüger *et al*., 2005). As such, it is commonly found in urban stormwater runoff with higher traffic volumes (Cao *et al*., 2022; Maurer *et al*., 2023; Liu et al., 2024a). 6PPD-Q was recently identified as the cause of acute mortality in Coho salmon in the Pacific Northwest and has since attracted considerable research attention to better understand its implications for other aquatic organisms (Tian *et al*., 2020). Phytoplankton monoculture responses to 6PPD-Q exposure have been mixed, with some species showing enhanced growth, others inhibited growth or hormetic responses, and alterations to photosynthesis (Liu et al., 2024b; Yan *et al*., 2024; You *et ali* 2024).

There is some evidence that exposure to these contaminants may affect community dynamics (Gomaa *et al*., 2021). However, much of the research on these contaminants has focused on phytoplankton monocultures and contaminant concentrations several orders of magnitude above environmentally relevant levels, thereby overlooking the potential sub-inhibitory effects on community composition at environmentally relevant concentrations. This project addresses that gap by investigating how a natural, representative freshwater phytoplankton community responds to exposure to these four contaminants at closer to environmentally relevant concentrations, thereby providing a more realistic assessment of their ecological impacts. Phytoplankton play invaluable roles in aquatic ecosystem processes and functions, including primary production, trophodynamics and biogeochemical cycling (Naselli-Flores and Padisák, 2023; Updhyay *et al*., 2025). Total community biomass, often measured as total chlorophyll *a* (chl *a*), can be an indicator of community health and potential productivity. However, community composition is a more valuable metric for assessing phytoplankton contributions in aquatic systems, as taxa may differ in functional traits and responses to perturbations (Litchman *et al*., 2007; Pritsch *et al*., 2023). Thus, evaluating responses of individual phytoplankton groups, in addition to community responses, provides valuable insights into implications for ecosystem health.

This study evaluates how exposure to four different CEC contaminants (carbamazepine, diclofenac, 6:2 FTS and 6PPD-Q) affect total biomass, community composition and photosynthetic capacity in a representative natural phytoplankton community. The objective was to determine the exposure levels (breakpoints) that cause sub-inhibitory adverse effects across different groups within the phytoplankton community. We conducted controlled microcosm bioassay experiments and used Bayesian segmented regression to estimate pollutant-specific ecological thresholds, providing a robust statistical framework for identifying nonlinear stressor–response relationships.

## METHODS

**Fig. 1:**
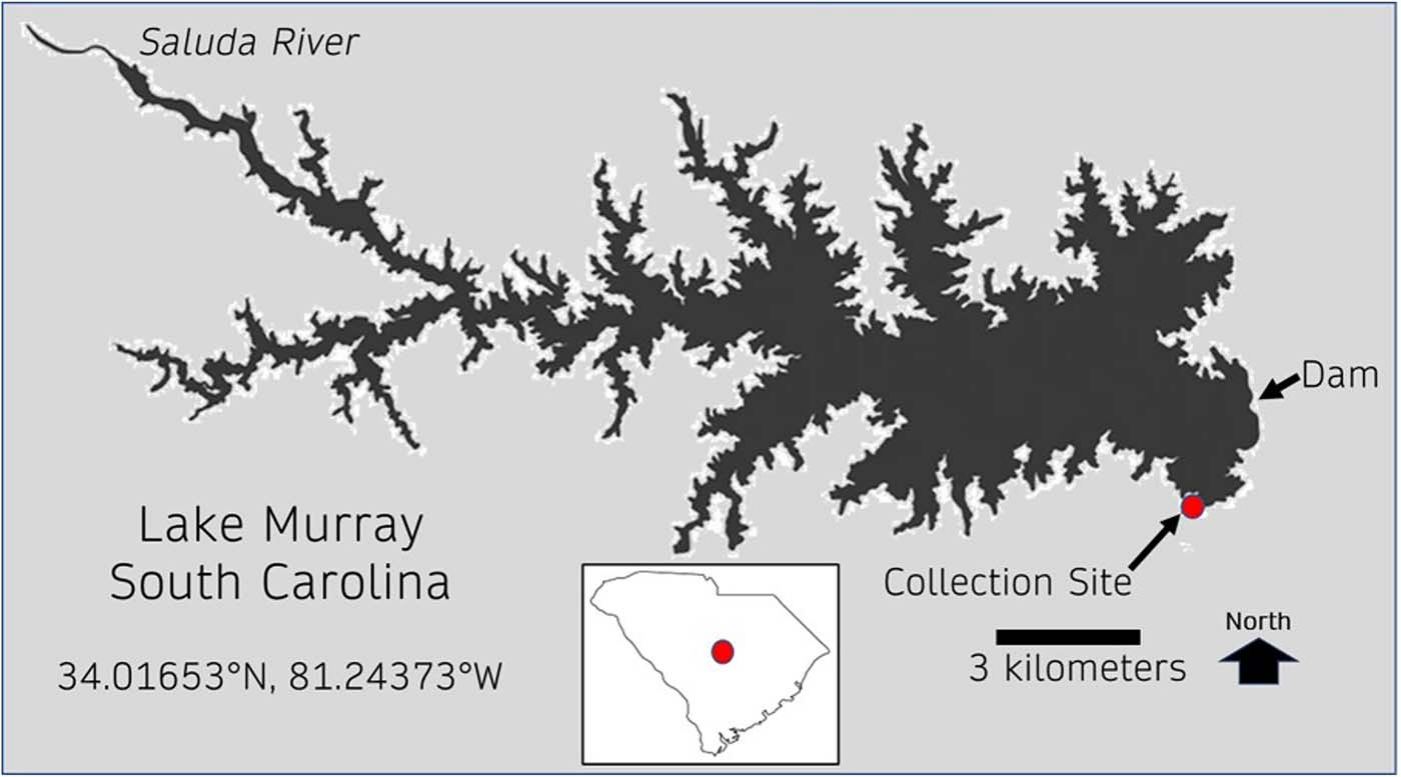
Lake Murray, South Carolina water collection site for phytoplankton bioassays.

Lake Murray, SC, has been the focus of ongoing phytoplankton monitoring since 2021. This lake is a managed hydroelectric reservoir (Dominion Energy) with an area of 182 km^2^, serves as a source for municipal water intakes and is a center for recreational activities. Weekly to fortnightly measurements from 2021 to 2023 indicated that the pooled relative contributions of phytoplankton groups during the summer months were: green algae (chlorophytes, euglenophytes and prasinophytes, 37%), diatoms (27%), cryptophytes (20%), cyanobacteria (11%) and dinoflagellates (4%) (Durbin *et al*., 2024, 2025). The phytoplankton community and all phytoplankton groups, except cyanobacteria, exhibit nutrient co-limitation, with N as the primary limiting nutrient. Similarly, cyanobacteria exhibit co-limitation, but with P as the primary nutrient (Durbin *et al*., 2025). Water samples were collected near the West Columbia municipal water intake (34.01653°N, 81.24373°W; Fig. 1) on 22 May 2024, 12 June 2024, 24 July 2024 and 21 August 2024, where each date represents the start of a separate bioassay for each contaminant (carbamazepine, diclofenac, 6:2 FTS and 6PPD-Q, respectively). These dates capture the major growing season for Lake Murray. On each date, a composite 20 L sample was collected from the surface waters. Water samples were transported to the lab for processing within 2 h.

### Contaminant concentrations

Ambient concentrations of 6:2 FTS and 6PPD-Q have previously been measured in this region of Lake Murray. In 2023, the South Carolina Department of Environmental Services reported non-detectable concentrations of 6:2 FTS, whereas other sites in South Carolina ranged from 1.26 to 127 ng L^−1^ (South Carolina Department of Environmental Services, n.d.). In 2024, concentrations of 6PPD-Q were consistently below detection limits (S. Richardson, pers. comm., 6 May 2025). Carbamazepine has not previously been measured in Lake Murray, but it has been measured elsewhere in South Carolina. In 2014, Bradley *et al.* (2016) found concentrations ranging from 0.8 to 11 ng L^−1^ in the upstate region and in 2019, Fahr *et al.* (2021) found carbamazepine concentrations up to ca. 50 ng L^−1^ in the Reedy River. To our knowledge, diclofenac concentrations in surface water have not been measured in South Carolina. These reported concentrations, as well as concentrations reported in Santos et al. (2010) and Meyer et al. (2016), were factored into the concentrations used for this study. While they are not fully reflective of previous measurements, the concentrations used in the bioassays (Table I) were chosen to be closer to environmentally relevant concentrations than those used in traditional ecotoxicology bioassays, which often span a wide range, and are often much higher than ambient concentrations. Further, because we were unable to measure background levels of contaminants or verify our dosing, contaminant levels should be considered nominal.

**Table I:** Summary information for the four contaminants used for the bioassays

	6:2 FTS	Carbamazepine	Diclofenac	6PPD-Q
Category	1H,1H,2H,2H-perfluorooctanesulfonic acid	Pharmaceutical	Pharmaceutical	6-PPD-quinone
Molecular formula	C_8_H_5_F_13_O_3_S	C_15_H_12_N_2_O	C_14_H_10_Cl_2_NNaO_2_	C_18_H_22_N_2_O_2_
Molecular weight (g mol^−1^)	428.17	236.27	318.13	298.38
CAS number	27619-97-2	298–46-4	15 307–79-6	2 754 428–18-5
Source	HPC Standards, Inc.	HPC Standards, Inc.	HPC Standards, Inc.	HPC Standards, Inc.
Target concentration (nM)	3	5	4	0.12
Incubation levels (nM)	0.00, 0.30, 0.75, 1.50, 2.25, 3.00, 3.75	0.00, 0.50, 1.25, 2.50 3.75, 5.00, 6.25	0.00, 0.40, 1.00, 2.00, 3.00, 4.00, 5.00	0.0057, 0.014, 0.029, 0.063, 0.10, 0.12, 0.14
Replicates per level	5	5	5	5
Incubation volume per replicate (mL)	850	250	250	850

### Bioassays

Before processing, a water subsample was filtered to measure initial phytoplankton biomass and community composition at time zero. The water was then divided into 250 mL culture flasks (for carbamazepine and diclofenac) or 850 mL culture flasks (for 6:2 FTS and 6PPD-Q) culture flasks, each receiving 10–125% of the target concentration for each contaminant, with five replicates per treatment level (Table I). The control treatment contained no added contaminants, and the solvent control received only the solvent carrier (acetone for carbamazepine and diclofenac; methanol for 6:2 FTS and 6PPD-Q). All bottles, including controls, received nutrient additions to achieve final concentrations of 20 μM N and 10 μM P (as NaNO_3_ and KH_2_PO_4_) to minimize nutrient limitation. Samples were incubated for 72 h under a 12:12 light cycle at room temperature (~24–26°C), gently mixed and rearranged under the light banks three times daily. Light was supplied by a 91 cm, 4 × 39 W Ocean Light T5 hood (10 000 K, 39 W—TRU fluorescent lamps) to achieve an irradiance of ~130 μmol photons m^−2^ s^−1^. After 72 h, samples were collected to measure biomass and community composition via high-performance liquid chromatography (HPLC), and photosynthetic efficiency (F_v_/F_m_) was measured using pulse-amplitude modulation (PAM) fluorometry. For HPLC analyses, 45–250 mL of water was vacuum filtered (−50 kPa) onto 2.5 cm glass microfiber filters (Sterlitech, 0.7 μm nominal pore size, GF/F) and stored at −80°C.

### Sample processing

HPLC analysis enabled quantification of chemotaxonomic photosynthetic pigments (Higgins *et al*., 2011). After lyophilization at −50°C for 24 h, samples were extracted in 90% acetone (750 μL) at −20°C for 24 h. The analysis used a Shimadzu 2050 HPLC system with two reverse-phase C-18 columns: a monomeric Rainin Microsorb-MV (0.46 × 10 cm, 3 μm) and a polymeric Vydac 201TP54 (0.46 × 25 cm, 5 μm). Pigments were separated using a nonlinear binary solvent gradient: 80% methanol:20% 0.50 M ammonium acetate and 80% methanol:20% acetone (Pinckney *et al*., 1996, 2001). After adding an ion-pairing agent (1.0 M ammonium acetate), filtered pigment extracts (250 μL) were injected, and their absorption spectra (380–700 nm) and chromatograms (440 ± 4 nm) were recorded with a photodiode array detector. Pigment peaks were identified by comparing retention times and absorption spectra with those of pure standards (DHI, Denmark). The synthetic carotenoid β-apo-8′-carotenal (Sigma) served as an internal standard. For details on the methodology, see Hooker *et al.* (2010).

We used CHEMTAX (version 1.95) to estimate the relative concentrations of major phytoplankton groups from photopigment concentrations (Pinckney *et al*., 2001; Higgins *et al*., 2011). This method quantifies the relative contributions of chl *a* to individual phytoplankton groups, including diatoms, green algae, cryptophytes, cyanobacteria, haptophytes and dinoflagellates. To minimize errors in biomass estimates arising from inaccurate initial pigment ratios, we applied the initial-ratio-matrix randomization technique with 60 matrices (Higgins *et al*., 2011). While this method misses finer-resolution species composition that may be used to determine species richness and diversity, it still informs about overall phytoplankton groups that may represent broader functional groups, a key metric of the phytoplankton community. The presence of such phytoplankton taxonomic groups was confirmed by qualitative microscopy at 400× magnification of randomly selected Lugol’s-fixed samples (2% final volume) using settling chambers and an inverted microscope. Within each bioassay, differences in total chl *a* between contaminant concentrations were determined via a one-way analysis of variance (ANOVA).

To measure photosynthetic efficiency (F_v_/F_m_), the basal fluorescence (F_o_) was recorded immediately after dark acclimation of the sample using a Walz Water PAM. This was followed by a saturating pulse of light (duration = 0.6 s; intensity 1200 μmol photons m^−2^ s^−1^) at which time the dark-adapted maximum fluorescence (F_m_) was recorded. Variable fluorescence (F_v_) is defined as the difference between maximum and basal fluorescence (F_m_–F_o_). Differences in F_v_/F_m_ between contaminant bioassays were evaluated using the Kruskal–Wallis test.

### Bayesian segmented regression

For each contaminant and algal group, chl *a* responses were modeled using a piecewise linear regression with a single unknown breakpoint (*τ*):


$${y}_i=\left\{\begin{array}{@{}cc}{\alpha}_L+{\beta}_L{x}_i+{\varepsilon}_i,& {x}_i\le \tau \\{}{\alpha}_R+{\beta}_R{x}_i+{\varepsilon}_i,& {x}_i>\tau \end{array}\right.$$

where ${y}_i$ denotes chl *a* concentration, ${x}_i$ denotes contaminant concentration and ${\varepsilon}_i\sim \mathcal{N}\left(0,{\sigma}_s^2\right)$ with segment-specific variance ${\sigma}_s^2$ for $s\in \left\{L,R\right\}$. We assumed noninformative priors for regression parameters, $p\left({\alpha}_s,{\beta}_s,{\sigma}_s^2\right)\propto 1/{\sigma}_s^2$, and a uniform prior over candidate breakpoints, $p\left(\tau \right)\propto 1$. Intercepts and slopes were estimated independently on either side of the breakpoint, allowing discontinuities in the mean response at *τ*. This permits responses that account for both abrupt ecological responses and the discrete contaminant concentrations used in these bioassays.

The breakpoint *τ* was treated as a discrete parameter and evaluated over a grid spanning the 5th to 95th percentiles of observed contaminant concentrations. The candidate breakpoint search was restricted to the 5th to 95th percentile of observed concentrations to prevent edge effects and ensure adequate data existed on both sides of each candidate breakpoint. For each candidate breakpoint, ordinary least squares regressions were fit separately to observations below and above *τ*. An approximate marginal likelihood was computed assuming normally distributed residuals. Posterior weights for candidate breakpoints were obtained by normalizing these likelihoods, yielding a discrete posterior distribution for *τ*.

Conditional on a given breakpoint, regression coefficients for each segment were sampled from conjugate normal posterior distributions centered on their least-squares estimates, with covariance proportional to the inverse Fisher information. Segment-specific residual variances were sampled from inverse-gamma distributions based on the residual sum of squares. Posterior samples were generated by first sampling a breakpoint from its discrete posterior distribution and then sampling regression parameters conditional on that breakpoint. Posterior medians and 95% credible intervals were used to summarize breakpoint locations and slope estimates for each algal group. All analyses were conducted in R (v. 4.5.2) using *MCMCpack* (Martin *et al*., 2011).

## RESULTS

**Fig. 2:**
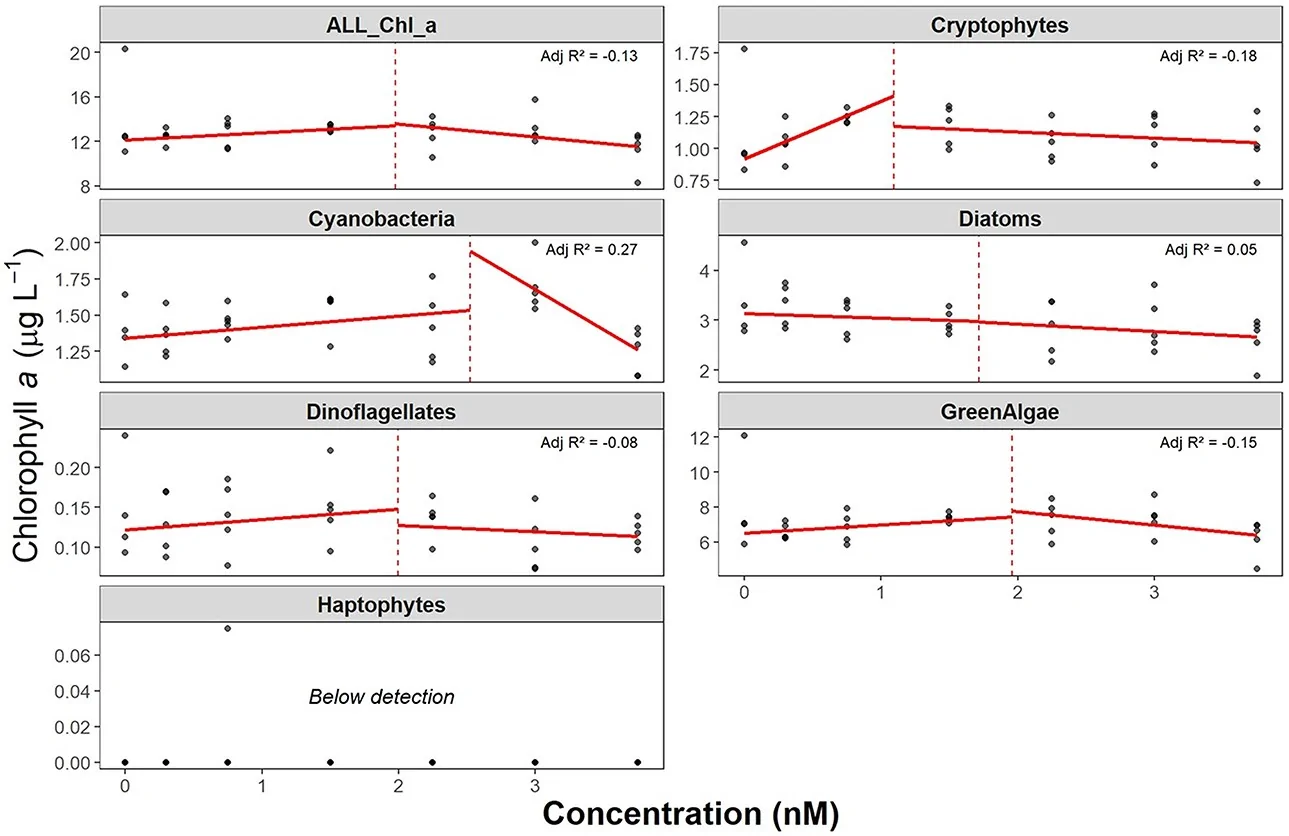
Chl *a* responses of phytoplankton algal groups to 6:2 FTS concentrations with Bayesian segmented regression fits. Dashed lines mark estimated breakpoints. Piecewise linear regression fits are shown using posterior median slopes and intercepts estimated independently on either side of the breakpoint, allowing for discontinuities at the threshold that capture abrupt ecological responses. Panels labeled “Below detection” indicate taxa for which no breakpoint could be estimated within the observed concentration range.

Bayesian segmented regression revealed several clear breakpoint-dependent changes in algal responses to 6:2 FTS exposure, with breakpoints ranging from 1.07 to 2.53 nM (Table II, Fig. 2, Supplementary Table S1). Cyanobacteria exhibited a positive slope below the breakpoint followed by a negative slope above it, with a posterior probability of 0.99 supporting a genuine shift in response direction for this algal group. Green algae and cryptophytes similarly showed positive slopes below the breakpoint followed by a negative slope above the breakpoint at higher 6:2 FTS concentrations. Total chl *a* exhibited a similar shift, with Bayesian credible intervals supporting a modest increase before the breakpoint and a decline thereafter. Dinoflagellates showed a weak but positive trend before the breakpoint, with little change in slope beyond it. In contrast, diatoms exhibited a negative slope both below and above the breakpoint, with the above-breakpoint decline slightly steeper, making them among the most 6:2 FTS-sensitive groups observed. Haptophytes remained below detection limits across all treatments and could not be modeled. Across algal groups, posterior slope estimates and their credible intervals indicate that 6:2 FTS effects are non-linear and exhibit threshold behavior, with several groups showing hormetic-like responses characterized by initial stimulation followed by inhibition once 6:2 FTS concentrations exceed the breakpoint.

**Table II:** Median breakpoint concentrations and change in slope (delta slope) of 6:2 FTS, 6PPD-Q, diclofenac and carbamazepine (nM) for major phytoplankton groups

Bayesian segmented regression summary			
*Median estimate (95% CrI)*			
Contaminant	Algal group	Breakpoint	Delta slope
6:2 FTS	All phytoplankton	1.96 (0.51, 2.92)	−1.95 (−4.80, −0.12)
	Cryptophytes	1.07 (0.41, 1.49)	−0.49 (−0.78, −0.22)
	Cyanobacteria	2.53 (1.15, 2.98)	−0.61 (−0.94, −0.12)
	Diatoms	1.73 (0.34, 2.92)	−0.16 (−2.32, 0.63)
	Dinoflagellates	2.00 (0.49, 2.96)	−0.02 (−0.09, 0.05)
	Green algae	1.96 (0.70, 2.94)	−1.34 (−3.04, 0.18)
	Haptophytes	BD	BD
6PPD-Q	All phytoplankton	0.06 (0.02, 0.10)	52.76 (−104.32, 450.99)
	Cryptophytes	0.03 (0.02, 0.10)	9.94 (−18.31, 33.26)
	Cyanobacteria	0.08 (0.02, 0.10)	2.62 (−8.82, 25.56)
	Diatoms	0.07 (0.02, 0.10)	8.05 (−45.66, 161.84)
	Dinoflagellates	0.03 (0.01, 0.10)	0.16 (−8.97, 5.38)
	Green algae	0.06 (0.02, 0.10)	29.13 (−40.04, 162.54)
	Haptophytes	0.05 (0.02, 0.09)	10.89 (−4.62, 26.75)
Diclofenac	All phytoplankton	0.98 (0.43, 2.89)	−1.10 (−7.90, 2.18)
	Cryptophytes	1.48 (0.45, 3.92)	−0.23 (−0.90, −0.04)
	Cyanobacteria	1.76 (0.43, 3.94)	0.12 (−0.04, 0.70)
	Diatoms	1.46 (0.43, 3.89)	−0.07 (−2.96, 2.07)
	Dinoflagellates	1.51 (0.45, 2.94)	−0.09 (−0.31, 0.02)
	Green algae	0.83 (0.43, 3.84)	−1.53 (−3.79, 0.10)
	Haptophytes	1.21 (0.43, 2.76)	0.08 (0.01, 0.20)
Carbamazepine	All phytoplankton	0.88 (0.50, 1.85)	−2.37 (−7.83, 2.67)
	Cryptophytes	1.85 (1.26, 2.45)	−0.77 (−1.07, −0.55)
	Cyanobacteria	2.61 (0.85, 3.74)	0.07 (−0.04, 0.23)
	Diatoms	0.88 (0.50, 1.76)	−1.28 (−5.21, 2.68)
	Dinoflagellates	2.45 (0.57, 4.84)	−0.02 (−0.07, 0.08)
	Green algae	0.91 (0.50, 3.52)	−0.06 (−1.09, 0.84)
	Haptophytes	1.70 (0.53, 4.21)	0.09 (−0.03, 0.28)

Lower values indicate greater sensitivity to contaminant exposure. “BD” denotes that the phytoplankton group abundance was below detection.

**Fig. 3:**
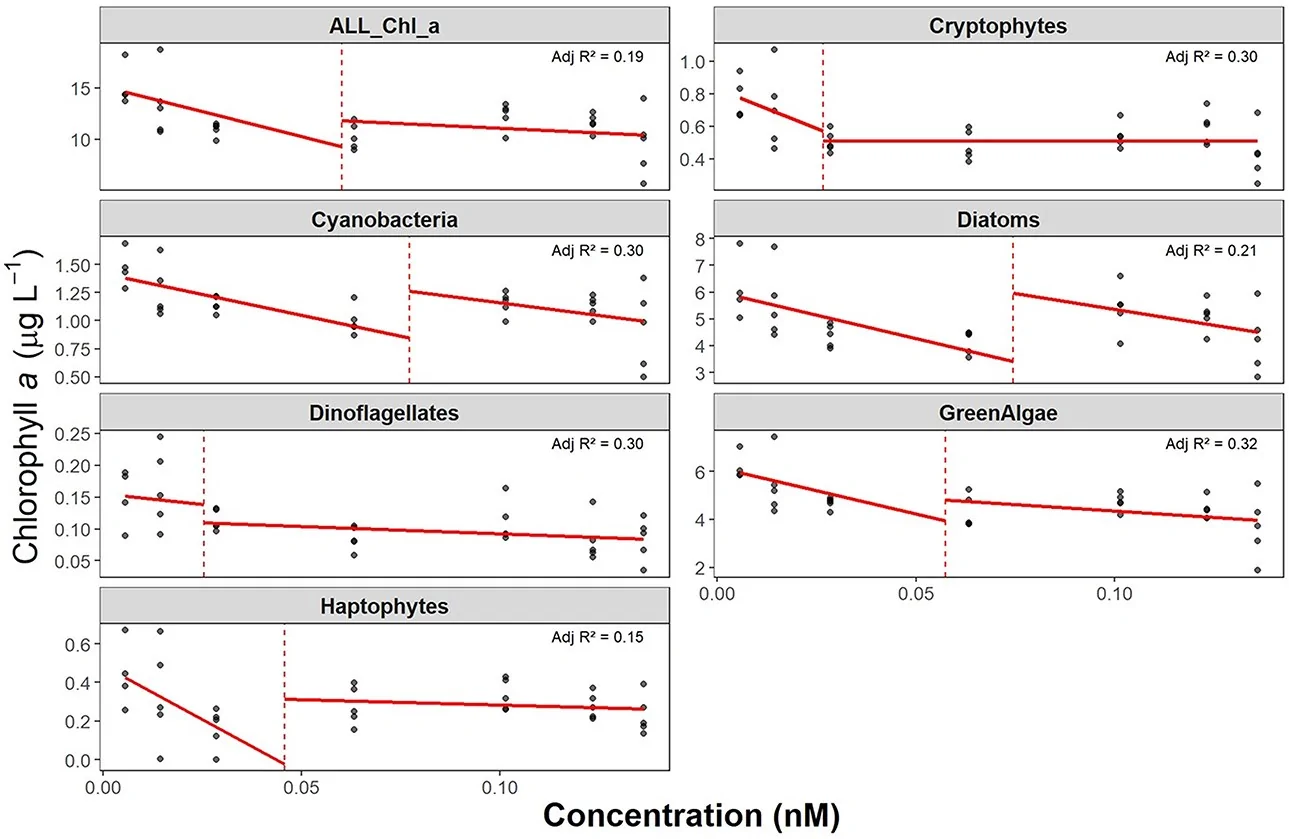
Chl *a* responses of phytoplankton algal groups to 6PPD-Q concentrations with Bayesian segmented regression fits. Dashed lines mark estimated breakpoints. Piecewise linear regression fits are shown using posterior median slopes and intercepts estimated independently on either side of the breakpoint, allowing for discontinuities at the threshold that capture abrupt ecological responses.

In the 6PPD-Q bioassays, cyanobacteria, cryptophytes, diatoms, haptophytes, green algae and total chl *a* showed negative slopes below the breakpoint, indicating similar responses in declines even at the lowest exposure concentrations (Table II, Fig. 3, Supplementary Table S2). Above the breakpoint, slopes generally flattened, and credible intervals overlapped at zero, suggesting reduced sensitivity or a leveling off of inhibitory effects at higher concentrations. Dinoflagellates exhibited weaker responses, with slopes both above and below the breakpoint characterized by wide credible intervals that included zero, indicating comparatively low responsiveness to 6PPD-Q. Breakpoint estimates were poorly constrained for many of the phytoplankton groups, with broad posterior intervals indicating uncertainty about the threshold location and the potential for multiple plausible breakpoint values.

**Fig. 4:**
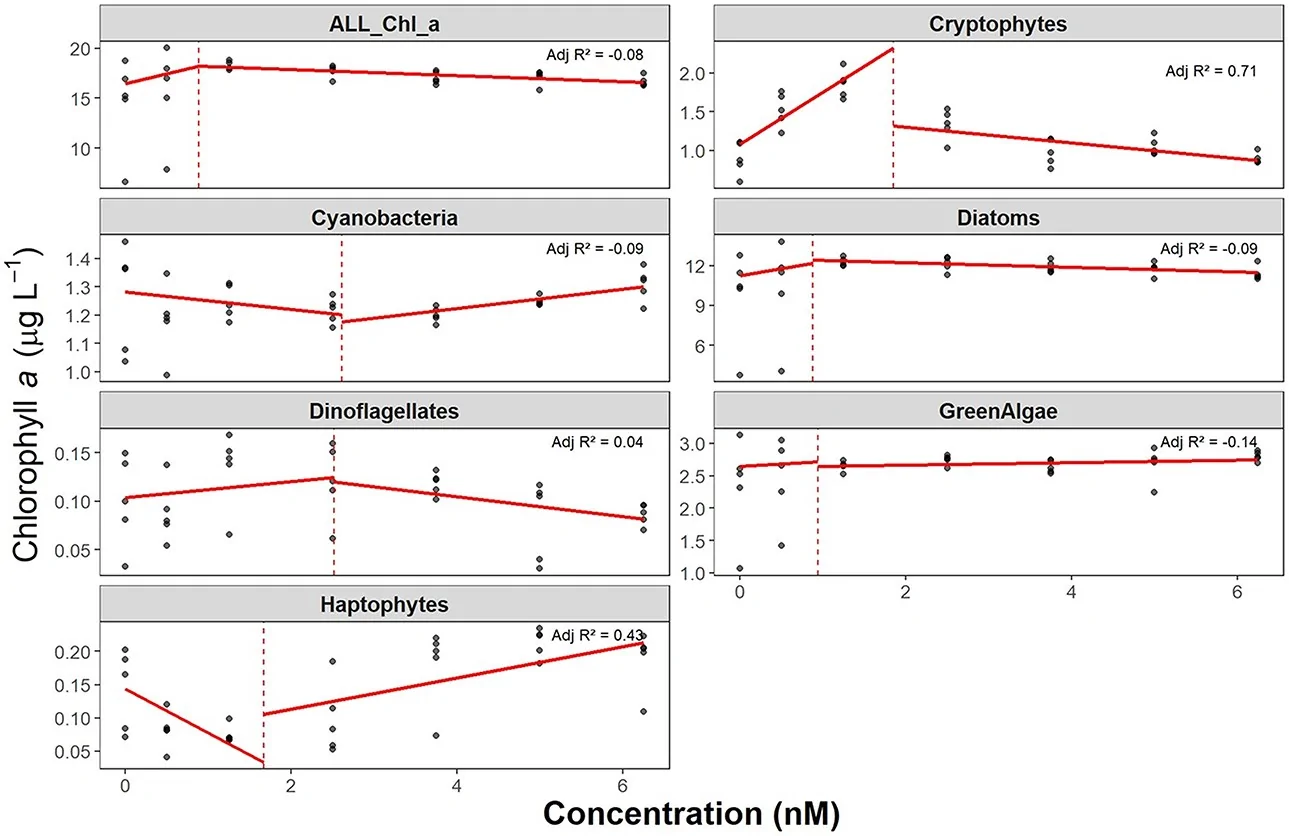
Chl *a* responses of phytoplankton algal groups to carbamazepine concentrations with Bayesian segmented regression fits. Dashed lines mark estimated breakpoints. Piecewise linear regression fits are shown using posterior median slopes and intercepts estimated independently on either side of the breakpoint, allowing for discontinuities at the threshold that capture abrupt ecological responses.

For carbamazepine, bioassay results showed weak, taxon-specific breakpoint responses (Table II, Fig. 4, Supplementary Table S3). Cyanobacteria exhibited slightly positive slopes above the breakpoint, with credible intervals that overlapped zero but suggesting modest increases in chl *a* at higher concentrations. Diatoms, dinoflagellates and green algae showed minimal change across the breakpoint, with below- and above-breakpoint slopes near zero and wide credible intervals, indicating limited sensitivity to carbamazepine. Cryptophytes and haptophytes displayed the most evident breakpoint patterns. Cryptophytes exhibited a positive slope below the breakpoint followed by a negative slope above it, whereas haptophytes exhibited a negative slope below the breakpoint, followed by a positive slope above it, with posterior probabilities of 0.93 for both. These patterns support genuine shifts in response direction. Total chl *a* declined slightly after the breakpoint, but credible intervals for both slopes included zero, indicating overall weak concentration dependence. Across taxa, posterior slope estimates indicate that carbamazepine effects were subtle, with most groups showing negligible or weakly negative responses. In contrast, distinct threshold-driven changes in sensitivity were evident only for cryptophytes and haptophytes.

**Fig. 5:**
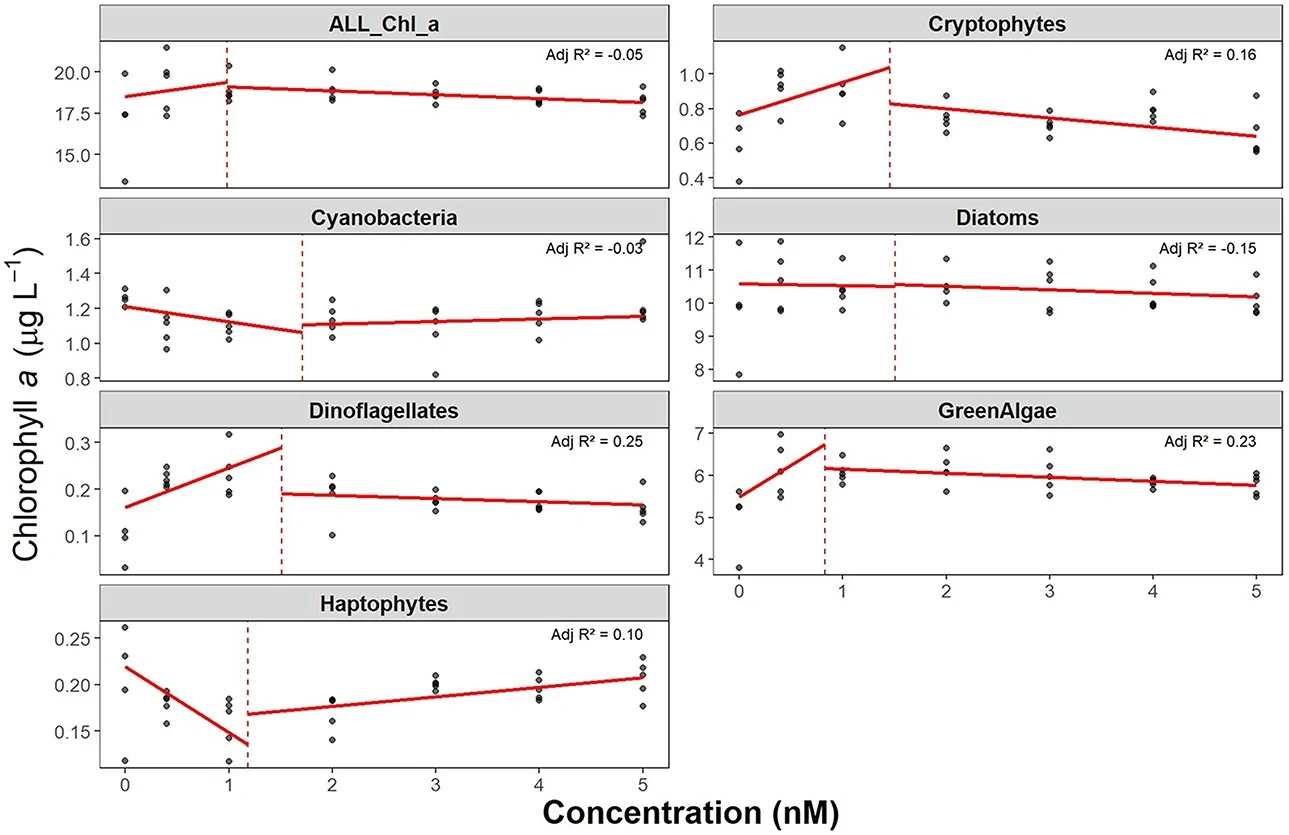
Chl *a* responses of phytoplankton algal groups to diclofenac concentrations with Bayesian segmented regression fits. Dashed lines mark estimated breakpoints. Piecewise linear regression fits are shown using posterior median slopes and intercepts estimated independently on either side of the breakpoint, allowing for discontinuities at the threshold that capture abrupt ecological responses.

The diclofenac bioassays indicated modest, phytoplankton group-specific threshold responses (Table II, Fig. 5, Supplementary Table S4). Cyanobacteria and diatoms exhibited consistently shallow negative slopes before and after the breakpoint, with posterior probabilities modestly favoring a negative trend but with wide credible intervals, suggesting mild but uncertain sensitivity. Dinoflagellates and cryptophytes showed positive slopes below the breakpoint, followed by a flattening of the above-breakpoint slope; nevertheless, the posterior probability of a change in slope direction was relatively high (0.91 and 0.99, respectively). In contrast, green algae and haptophytes displayed the clearest breakpoint behavior. Green algae exhibited a positive slope below the breakpoint, and a negative slope above it, whereas haptophytes exhibited a negative slope below the breakpoint, followed by a positive slope above it. Posterior probabilities were 0.95 and 0.99, respectively, and support genuine shifts in response direction. Total chl *a* declined slightly above the breakpoint, but credible intervals for both segments included zero, reflecting minimal concentration dependence overall. Across phytoplankton groups, posterior slope estimates indicated that diclofenac effects were subtle: most responses were characterized by shallow slopes and high uncertainty, and distinct directional changes across the breakpoint were evident only for green algae and haptophytes.

**Fig. 6:**
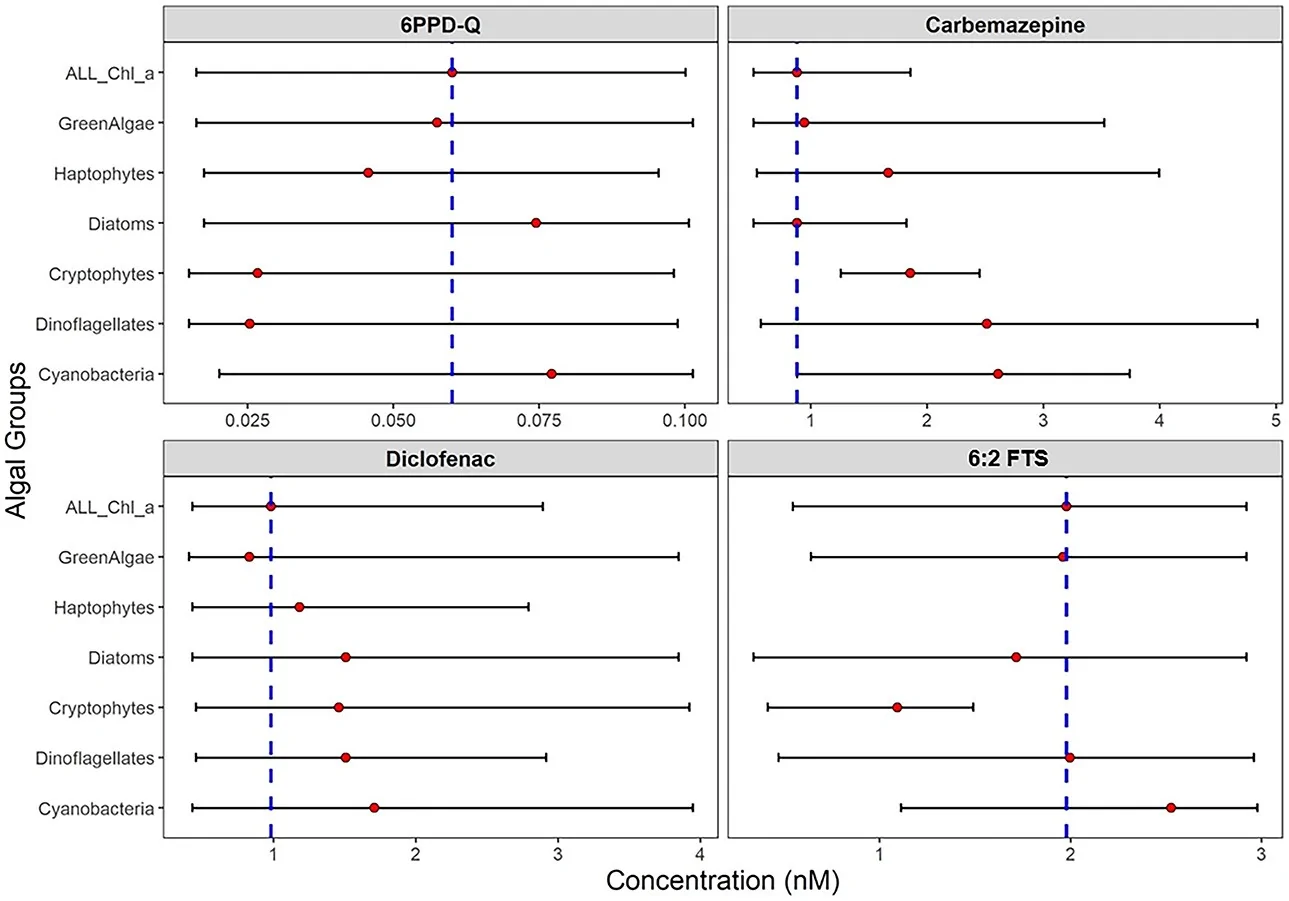
Bayesian segmented breakpoint estimates for individual algal groups compared against the weighted phytoplankton community breakpoint (vertical dashed line) for each contaminant. Points and intervals show the variability (95% credible intervals) in taxon-specific thresholds.

Breakpoint estimates revealed distinct contaminant-specific thresholds in phytoplankton sensitivity (Fig. 6). Across all four CECs, algal groups showed broad but overlapping ranges of breakpoint concentrations, with credible intervals indicating substantial variability in taxon-level responses. For diclofenac, breakpoint estimates for most phytoplankton groups clustered just above the community-level threshold, suggesting relatively consistent sensitivity across taxa. In contrast, 6:2 FTS breakpoints were more widely dispersed, with several groups showing lower thresholds than the overall community response, indicating heightened sensitivity among specific phytoplankton groups, particularly cryptophytes. Carbamazepine produced low breakpoint values relative to the total range of incubation concentrations with comparatively smaller credible intervals and good posterior probability of a change in slope direction for several taxa (e.g. diatoms, cryptophytes, haptophytes). These are consistent with weak concentration dependence and limited divergence among algal groups relative to the community estimate (total chl *a*). Compared to the other contaminants, 6PPD-Q produced the highest and most variable breakpoint estimates, with several algal groups responding at concentrations below the phytoplankton community threshold, consistent with its stronger inhibitory effects at low exposure levels. Overall, these patterns indicate that chemical identity strongly influences both the magnitude and variability of phytoplankton breakpoint responses, with some contaminants eliciting uniform thresholds across taxa, whereas others elicit highly heterogeneous sensitivity patterns.

**Fig. 7:**
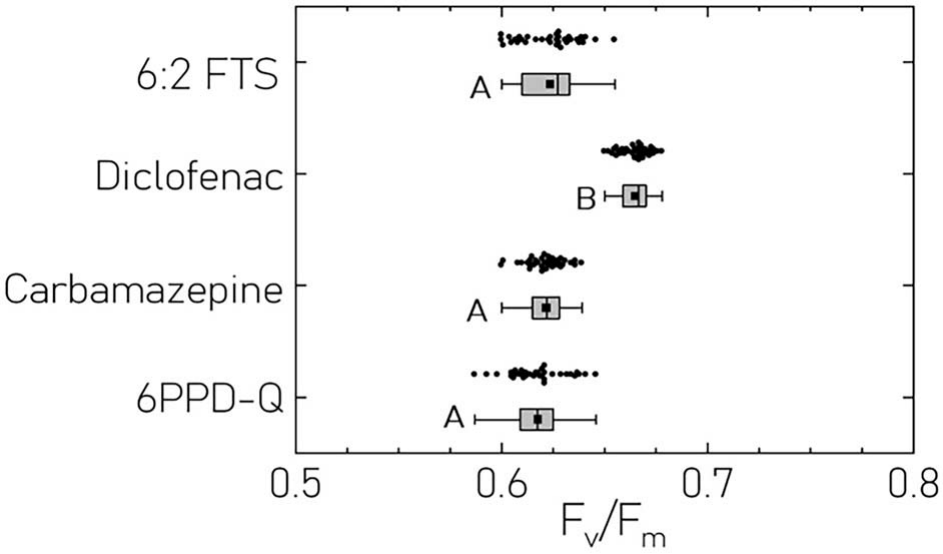
Boxplots of maximum photochemical efficiency (F_v_/F_m_) for each bioassay. Means are indicated by small squares within the box, and medians by a line within the box. The box indicates the first and third quartiles, and the bars show the 5th and 95th percentiles. Letters next to the boxplots indicate the outcomes of post hoc comparisons.

**Fig. 8:**
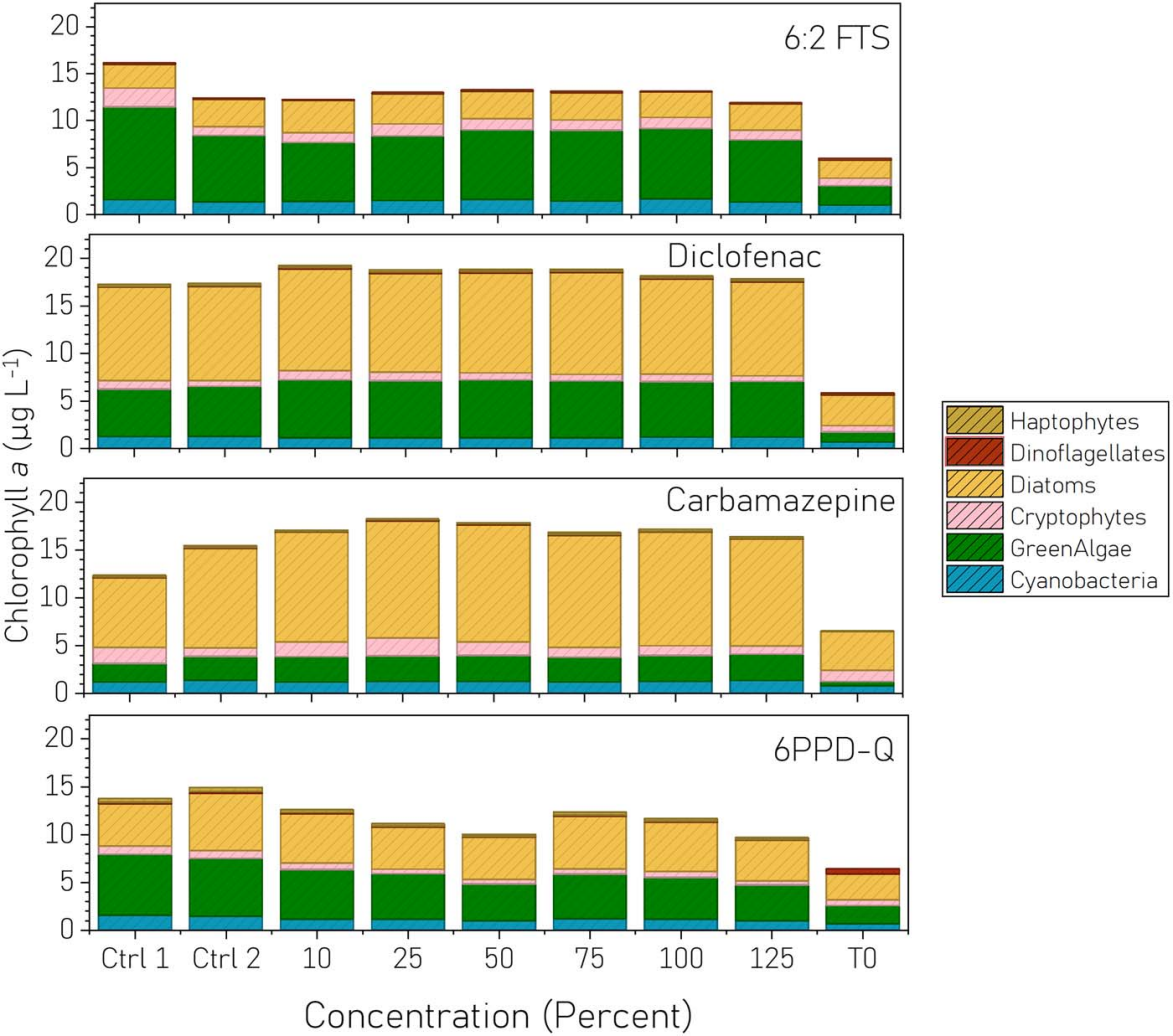
Stacked bar graph of phytoplankton group biomass (as μg L^−1^ chl *a*) at 72 h based on the median of five replicates. Concentration is indicated as a percentage of the target concentration (Table I). “Ctrl 1” received no additions other than nutrients; “Ctrl 2” received nutrients and the relevant solvent only.

Across all treatments, maximum photochemical efficiency (F_v_/F_m_) and total community chl *a* did not show compound-specific responses in the bioassays (Figs 7 and 8). Variation in F_v_/F_m_ within each bioassay was minimal (coefficient of variation ranged from 0.1 to 0.2), suggesting no effect of exposure level within each bioassay. Differences between the four bioassays could be attributed to differences in the initial phytoplankton communities. Although the diclofenac bioassay had a higher median F_v_/F_m_ than the others (Kruskal–Wallis, χ^2^ = 78.03, *P* < 0.001), the difference is unlikely to be ecologically relevant.

## DISCUSSION

The pattern of total community biomass remaining largely unchanged across concentrations of carbamazepine, diclofenac and 6:2 FTS aligns with EC_50_ values for these contaminants, which vary by species but generally fall within the moderate- to low-concern ranges under the EPA hazard ranking system (U.S. Environmental Protection Agency [EPA], 1996; Cleuvers, 2004; DeLorenzo and Fleming, 2008; Zhang *et al*., 2012; Zhang *et al*., 2023). This result is also consistent with other studies examining the effects of contaminants on freshwater communities (Versteegen *et al*., 2025). Additionally, photosynthetic capacity was unaffected by exposure to any of the four CECs. However, these community-level parameters do not reveal potential taxa-specific responses to CEC exposure, which may be necessary for primary productivity and other ecosystem services provided by the phytoplankton community.

The breakpoint patterns observed across contaminants may indicate fundamentally different modes of action that influence phytoplankton sensitivity and subsequent alterations in community composition, though they may also potentially represent differences in initial phytoplankton communities. 6:2 FTS produced breakpoints that generally occurred at or below the total community breakpoint, and similar negative responses above the breakpoint were observed across algal groups. This pattern is consistent with a mode of action that broadly disrupts membrane integrity and induces oxidative damage, disrupts ion regulation or affects intracellular signaling, thereby affecting many taxa similarly, as is common among other PFAS (Ma *et al*., 2022). This uniformity suggests that 6:2 FTS exposure acts as a general metabolic stressor rather than targeting any specific physiological pathway. This somewhat aligns with the relatively similar EC_50_ values for 6:2 FTS across species within different groups: >125 mg L^−1^  *P. subcapitata* (72 h EC_50_; Hoke *et al*., 2015), 314.8 mg L^−1^ for *C. vulgaris* (4 day EC_50_; Zhang *et al*., 2023); and 317.5 mg L^−1^ for *M. aeruginosa* (4 day EC_50_; Zhang *et al*., 2023). The lack of a response in F_v_/F_m_ to 6:2 FTS exposure aligns with previous studies that have found little impact on F_v_/F_m_ in *C. vulgaris* and *M. aeruginosa* at concentrations below 1 mg L^−1^, though high concentrations (>20 mg L^−1^) may lead to steep declines in F_v_/F_m_ (Zhang *et al*., 2023).

Similarly, carbamazepine produced uniformly weak responses with low breakpoints, suggesting limited cellular uptake or a mode of action that interacts minimally with phytoplankton primary metabolic pathways. These results align with evidence that carbamazepine is persistent but not strongly phytotoxic at environmentally relevant concentrations, with lowest observed effect concentration (LOEC) and EC_50_ values across species that are relatively similar (74–80 mg L^−1^; Cleuvers, 2003; Ferrari *et al*., 2003; DeLorenzo and Fleming, 2008; Versteegen *et al*., 2025). This pattern could also reflect the duration of exposure, as some studies suggest that carbamazepine has a greater impact on phytoplankton under chronic exposure than under acute exposure (Zhang *et al*., 2012). However, it is worth considering that this may reflect limited interaction with the contaminant. There is evidence that carbamazepine adsorbs to plastic materials, potentially reducing its availability (Griffin and D'Arcy, 1997). Unfortunately, measuring dissolved carbamazepine concentrations was outside the scope of this project; therefore, the impacts of carbamazepine warrant further exploration.

In contrast, diclofenac produced more clustered breakpoints with wide credible intervals, suggesting a mechanism involving interference with photosynthesis and oxidative balance, processes that are broadly conserved among phytoplankton, but with some taxa-specific differences (Cirulis *et al*., 2013; Majewska *et al*., 2018; McCain and Bertrand, 2022; Krawczyk *et al*., 2025). This uniformity contrasts with studies demonstrating varying sensitivity to diclofenac among phytoplankton species and groups, thereby affecting diversity and community composition; however, these studies either considered cell counts rather than biomass or lacked representation across algal groups (Duarte *et al*., 2023; Nugnes *et al*., 2025). Alternatively, this could again be an artifact of group-level generalization from photopigment analysis, obscuring major changes in species-specific dynamics, yet still informing general functional dynamics, which would be consistent with the specific differences noted in Nugnes *et al.* (2025).

6PPD-Q showed the highest and most variable breakpoints, aligning with its known capacity to cause oxidative stress through quinone redox cycling (You *et al*., 2024). Quinones can undergo repeated electron transfer, producing reactive oxygen species (ROS). The noticeable early declines in chl *a*, along with varying taxon-level thresholds, indicate significant differences among taxa in their antioxidant capacity, redox-buffering systems and vulnerability to ROS-induced damage (Yan *et al*., 2024). Variations in ROS production and exposure, combined with distinct antioxidant defenses, result in considerable differences in susceptibility to oxidative harm from 6PPD-Q, explaining the high variability in breakpoints (Passardi *et al*., 2007; Diaz and Plummer, 2018; Omar *et al*., 2023). This is supported by studies showing species-specific responses to 6PPD-Q, although a general pattern of hormetic response is observed (Liu et al., 2024b; Yan *et al*., 2024).

From a management perspective, these mechanistic differences significantly impact environmental monitoring and setting regulatory thresholds. Contaminants like 6:2 FTS and diclofenac, which cause consistent or weak responses at the taxon level, can be reasonably monitored through community-wide metrics (such as total chl *a* or aggregated community breakpoints), since these metrics approximate the distribution of sensitivities across groups. In contrast, carbamazepine and, especially, 6PPD-Q pose significant management challenges because their effects are highly specific to certain taxa. These substances can alter community composition well before affecting overall biomass, often by disproportionately suppressing sensitive groups such as diatoms or cryptophytes. Such changes can affect nutrient cycling, food-web structure and competitive interactions, ultimately influencing ecosystem function even when community-level indicators (e.g. total chl *a*) appear unchanged. This is especially relevant in locations with different species assemblages and dominant groups that may vary in sensitivity to these contaminants. Further, some of the wide credible intervals could indicate species-specific differences in responses that are not visible with HPLC, highlighting the need for future work investigating species composition at a finer taxonomic resolution.

The wide uncertainty intervals and taxon-specific divergence observed for carbamazepine and 6PPD-Q underscore the need for multi-taxa monitoring frameworks rather than relying solely on total chl *a* or bulk community responses. Moreover, the steep below-breakpoint declines in several contaminants suggest that regulatory thresholds should extend beyond sub-lethal effects to encompass ecologically significant early-stage impacts, including altered growth trajectories, oxidative stress and shifts in competitive dynamics. Incorporating Bayesian breakpoint models into routine risk assessment provides a transparent, probabilistic means of defining these early-warning thresholds.

Overall, the results indicate that effective management of emerging contaminants should adopt contaminant-specific monitoring strategies. Although community-level thresholds may suffice for broadly acting compounds, chemicals with taxon-specific or redox-active modes of action require more nuanced, taxa-resolved measurements to detect shifts in community structure and function.

## CONCLUSION

The breakpoint analyses show that emerging contaminants differ markedly in how they disrupt phytoplankton communities, with some exerting consistent, community-wide effects and others selectively affecting vulnerable taxa. These contrasting patterns of contaminant responses underscore that total biomass (i.e. chl *a*) alone cannot fully capture contaminant-induced shifts in phytoplankton community structure. Chemicals that act through broad disruptive mechanisms, such as 6:2 FTS, may be effectively monitored using community-level thresholds. In contrast, compounds with taxon-specific or oxidative modes of action (particularly 6PPD-Q) require more nuanced, multi-taxa assessments to detect early ecological impacts. By integrating Bayesian breakpoints with mechanistic insights, this work emphasizes the need for contaminant-specific monitoring strategies that account for both community-level responses and hidden shifts in taxonomic composition. Such an approach will enhance the ecological relevance of water-quality guidelines and help safeguard primary producers that support numerous critical processes in aquatic ecosystems.

## Supplementary Material

Supplementary_Information_fbag072

## Data Availability

Data are available upon request to the senior author.
