# Supplementary material for: Phytoplankton community responses to sub-inhibitory levels of four contaminants of emerging concern
Source: J Plankton Res. 2026 Aug 26;48(5):fbag072. doi: 10.1093/plankt/fbag072 (PMC13518054; doi:10.1093/plankt/fbag072)
Supplement: Supplementary file 1 [file supplementary_information_fbag072.docx]

**Supplementary Data**

**Tables**

***Table S1.*** *Median breakpoint concentrations, slope below the breakpoint (Left slope), slope above the breakpoint (Right slope), change in slope (Delta slope), directions of slope change (Direction), and posterior probability of slope change (P(direction)) of 6:2 FTS (nM) for major phytoplankton groups. “BD” denotes that the group abundance was below detection. Lower breakpoint values indicate greater sensitivity to contaminant exposure. Higher posterior probabilities indicate greater likelihood of a response.*

|  | **Bayesian segmented regression summary** | | | | | |
| --- | --- | --- | --- | --- | --- | --- |
|  | *Median estimate (95% CrI); P(delta ≠ 0) is posterior probability of slope change* | | | | | |
|  | **6:2 FTS** | | | | | |
| **Algal group** | **Breakpoint** | **Left slope** | **Right slope** | **Delta slope** | **Direction** | **P(direction)** |
| All phytoplankton | 1.96 (0.51, 2.92) | 0.64 (-0.15, 1.93) | -1.15 (-4.28, 0.28) | -1.95 (-4.80, -0.12) | Decrease | 0.981 |
| Cryptophytes | 1.07 (0.41, 1.49) | 0.45 (0.18, 0.70) | -0.05 (-0.14, 0.04) | -0.49 (-0.78, -0.22) | Decrease | 0.998 |
| Cyanobacteria | 2.53 (1.15, 2.98) | 0.07 (-0.02, 0.23) | -0.55 (-0.86, 0.00) | -0.61 (-0.94, -0.12) | Decrease | 0.993 |
| Diatoms | 1.73 (0.34, 2.92) | -0.09 (-0.58, 2.20) | -0.14 (-0.92, 0.27) | -0.16 (-2.32, 0.63) | Decrease | 0.661 |
| Dinoflagellates | 2.00 (0.49, 2.96) | 0.01 (-0.03, 0.08) | -0.01 (-0.03, 0.05) | -0.02 (-0.09, 0.05) | Decrease | 0.697 |
| Green Algae | 1.96 (0.70, 2.94) | 0.49 (-0.35, 1.11) | -0.78 (-2.60, 0.13) | -1.34 (-3.04, 0.18) | Decrease | 0.966 |
| Haptophytes | BD | BD | BD | BD | BD | BD |

***Table S2.*** *Median breakpoint concentrations, slope below the breakpoint (Left slope), slope above the breakpoint (Right slope), change in slope (Delta slope), directions of slope change (Direction), and posterior probability of slope change (P(direction)) of 6PPD-Q (nM) for major phytoplankton groups. “BD” denotes that the group abundance was below detection. Lower breakpoint values indicate greater sensitivity to contaminant exposure. Higher posterior probabilities indicate greater likelihood of a response.*

|  | **Bayesian segmented regression summary** | | | | | |
| --- | --- | --- | --- | --- | --- | --- |
|  | *Median estimate (95% CrI); P(delta ≠ 0) is posterior probability of slope change* | | | | | |
|  | **6PPD-Q** | | | | | |
| **Algal group** | **Breakpoint** | **Left slope** | **Right slope** | **Delta slope** | **Direction** | **P(direction)** |
| All phytoplankton | 0.06 (0.02, 0.10) | -96.31 (-453.36, 31.76) | -21.34 (-136.75, 22.34) | 52.76 (-104.32, 450.99) | Increase | 0.730 |
| Cryptophytes | 0.03 (0.02, 0.10) | -9.97 (-33.01, 17.56) | -0.04 (-5.09, 1.60) | 9.94 (-18.31, 33.26) | Increase | 0.822 |
| Cyanobacteria | 0.08 (0.02, 0.10) | -7.58 (-25.95, -2.39) | -4.36 (-14.28, 2.56) | 2.62 (-8.82, 25.56) | Increase | 0.682 |
| Diatoms | 0.07 (0.02, 0.10) | -35.36 (-156.59, 4.65) | -23.50 (-63.93, 13.91) | 8.05 (-45.66, 161.84) | Increase | 0.617 |
| Dinoflagellates | 0.03 (0.01, 0.10) | -0.83 (-5.56, 8.76) | -0.23 (-1.54, 0.25) | 0.16 (-8.97, 5.38) | Increase | 0.532 |
| Green Algae | 0.06 (0.02, 0.10) | -39.38 (-166.98, -2.80) | -10.72 (-54.51, 5.34) | 29.13 (-40.04, 162.54) | Increase | 0.745 |
| Haptophytes | 0.05 (0.02, 0.09) | -11.43 (-26.69, 2.70) | -0.54 (-4.01, 1.28) | 10.89 (-4.62, 26.75) | Increase | 0.890 |

***Table S3.*** *Median breakpoint concentrations, slope below the breakpoint (Left slope), slope above the breakpoint (Right slope), change in slope (Delta slope), directions of slope change (Direction), and posterior probability of slope change (P(direction)) of carbamazepine (nM) for major phytoplankton groups. “BD” denotes that the group abundance was below detection. Lower breakpoint values indicate greater sensitivity to contaminant exposure. Higher posterior probabilities indicate greater likelihood of a response.*

|  | **Bayesian segmented regression summary** | | | | | |
| --- | --- | --- | --- | --- | --- | --- |
|  | *Median estimate (95% CrI); P(delta ≠ 0) is posterior probability of slope change* | | | | | |
|  | **Carbamazepine** | | | | | |
| **Algal group** | **Breakpoint** | **Left slope** | **Right slope** | **Delta slope** | **Direction** | **P(direction)** |
| All phytoplankton | 0.88 (0.50, 1.85) | 2.08 (-2.96, 7.50) | -0.30 (-0.43, -0.16) | -2.37 (-7.83, 2.67) | Decrease | 0.847 |
| Cryptophytes | 1.85 (1.26, 2.45) | 0.67 (0.46, 0.95) | -0.10 (-0.17, -0.05) | -0.77 (-1.07, -0.55) | Decrease | 1.000 |
| Cyanobacteria | 2.61 (0.85, 3.74) | -0.03 (-0.21, 0.06) | 0.03 (0.01, 0.06) | 0.07 (-0.04, 0.23) | Increase | 0.932 |
| Diatoms | 0.88 (0.50, 1.76) | 1.09 (-2.86, 5.01) | -0.17 (-0.28, -0.07) | -1.28 (-5.21, 2.68) | Decrease | 0.761 |
| Dinoflagellates | 2.45 (0.57, 4.84) | 0.01 (-0.09, 0.06) | -0.01 (-0.02, 0.02) | -0.02 (-0.07, 0.08) | Decrease | 0.753 |
| Green Algae | 0.91 (0.50, 3.52) | 0.08 (-0.82, 1.11) | 0.02 (-0.02, 0.08) | -0.06 (-1.09, 0.84) | Decrease | 0.563 |
| Haptophytes | 1.70 (0.53, 4.21) | -0.07 (-0.25, 0.01) | 0.02 (-0.02, 0.04) | 0.09 (-0.03, 0.28) | Increase | 0.932 |

***Table S4.*** *Median breakpoint concentrations, slope below the breakpoint (Left slope), slope above the breakpoint (Right slope), change in slope (Delta slope), directions of slope change (Direction), and posterior probability of slope change (P(direction)) of diclofenac (nM) for major phytoplankton groups. “BD” denotes that the group abundance was below detection. Lower breakpoint values indicate greater sensitivity to contaminant exposure. Higher posterior probabilities indicate greater likelihood of a response.*

|  | **Bayesian segmented regression summary** | | | | | |
| --- | --- | --- | --- | --- | --- | --- |
|  | *Median estimate (95% CrI); P(delta ≠ 0) is posterior probability of slope change* | | | | | |
|  | **Diclofenac** | | | | | |
| **Algal group** | **Breakpoint** | **Left slope** | **Right slope** | **Delta slope** | **Direction** | **P(direction)** |
| All phytoplankton | 0.98 (0.43, 2.89) | 0.83 (-2.36, 7.68) | -0.24 (-0.53, -0.02) | -1.10 (-7.90, 2.18) | Decrease | 0.818 |
| Cryptophytes | 1.48 (0.45, 3.92) | 0.19 (-0.08, 0.85) | -0.05 (-0.25, -0.00) | -0.23 (-0.90, -0.04) | Decrease | 0.987 |
| Cyanobacteria | 1.76 (0.43, 3.94) | -0.08 (-0.69, 0.01) | 0.01 (-0.06, 0.09) | 0.12 (-0.04, 0.70) | Increase | 0.916 |
| Diatoms | 1.46 (0.43, 3.89) | -0.06 (-2.13, 2.88) | -0.10 (-0.51, 0.25) | -0.07 (-2.96, 2.07) | Decrease | 0.554 |
| Dinoflagellates | 1.51 (0.45, 2.94) | 0.08 (-0.02, 0.30) | -0.01 (-0.02, 0.01) | -0.09 (-0.31, 0.02) | Decrease | 0.912 |
| Green Algae | 0.83 (0.43, 3.84) | 1.44 (-0.09, 3.66) | -0.09 (-0.25, 0.06) | -1.53 (-3.79, 0.10) | Decrease | 0.953 |
| Haptophytes | 1.21 (0.43, 2.76) | -0.07 (-0.19, -0.01) | 0.01 (-0.00, 0.02) | 0.08 (0.01, 0.20) | Increase | 0.995 |
